# COVID-19 Spatio-Temporal Evolution Using Deep Learning at a European Level

**DOI:** 10.3390/s22103658

**Published:** 2022-05-11

**Authors:** Ioannis Kavouras, Maria Kaselimi, Eftychios Protopapadakis, Nikolaos Bakalos, Nikolaos Doulamis, Anastasios Doulamis

**Affiliations:** School of Rural, Surveying and Geoinformatics Engineering, National Technical University of Athens, 15772 Athens, Greece; eftprot@mail.ntua.gr (E.P.); bakalosnik@mail.ntua.gr (N.B.); ndoulam@cs.ntua.gr (N.D.); adoulam@cs.ntua.gr (A.D.)

**Keywords:** COVID-19 policies, deep learning, time-series prediction, COVID-19 reported cases, data-driven pandemic interventions

## Abstract

COVID-19 evolution imposes significant challenges for the European healthcare system. The heterogeneous spread of the pandemic within EU regions elicited a wide range of policies, such as school closure, transport restrictions, etc. However, the implementation of these interventions is not accompanied by the implementation of quantitative methods, which would indicate their effectiveness. As a result, the efficacy of such policies on reducing the spread of the virus varies significantly. This paper investigates the effectiveness of using deep learning paradigms to accurately model the spread of COVID-19. The deep learning approaches proposed in this paper are able to effectively map the temporal evolution of a COVID-19 outbreak, while simultaneously taking into account policy interventions directly into the modelling process. Thus, our approach facilitates data-driven decision making by utilizing previous knowledge to train models that predict not only the spread of COVID-19, but also the effect of specific policy measures on minimizing this spread. Global models at the EU level are proposed, which can be successfully applied at the national level. These models use various inputs in order to successfully model the spatio-temporal variability of the phenomenon and obtain generalization abilities. The proposed models are compared against the traditional epidemiological and Autoregressive Integrated Moving Average (ARIMA) models.

## 1. Introduction

At the end of 2019, China reported to World Health Organization (WHO) several cases of unusual pneumonia [1]. On January, 2020, COVID-19 (Coronavirus disease 2019 caused by Sars-Cov-2) was identified as a new disease and announced to the public, and by the end of January, WHO had declared a worldwide health emergency. By the following weeks, COVID-19 was spread to several countries around the globe. By the end of March 2019, the epidemic had already turned into a pandemic [2], and most countries were applying restrictive measures, such as lockdowns [3], as over 1,000,000 cases and more than 50,000 deaths were reported worldwide [4]. Nowadays, the national health agencies have developed response plans and tools for fighting the pandemic crisis. The COVID-19 outbreak demonstrates a significant heterogeneity on how it has been spread over different EU regions. This heterogeneity is mostly attributed to the management of the health crisis, including the way that regional authorities impose and monitor implementation of restrictions. The efficacy of the imposed restrictive measures, such as social isolation and lockdowns, has been evaluated worldwide and seems to vary significantly based on: (i) the strictness of government policies and (ii) the monitoring of their implementation. Classical epidemiological modelling of COVID-19 outbreaks, i.e. the calculation and mapping of the virus’ reproduction rate, has been an effective tool in monitoring similar past outbreaks, but mostly works when the spatial granularity of the analysis is small enough to disregard other socio-economic parameters.

Beyond the classical epidemiological approach, different time series predictive models have been applied in the literature to tackle the pandemic situation. Most of these proposed models exploit the temporal dynamics of the pandemic and suggest auto-regressive models to model the situation. However, it is noted that the time series trend for the infection differs between the countries depending on the strategies adopted by the healthcare organizations to decrease the spread. In particular, the unprecedented infection rate of COVID-19 led local authorities worldwide to adopt multiple and varying mitigation strategies as: (a) school closures; (b) workplace closures; (c) cancelling public events; (d) restrictions in gatherings; (e) closing public transportation; (f) stay-at-home requirements; (g) public information campaigns; (h) restrictions in internal movements; (i) international travel controls; and (j) facial coverings. These preventive measures vary per country, per severance level, and per time duration. Each country imposes a different set of measures that affect the transmission rate of the COVID-19 to a different extent. Thus, there is a need to create a COVID-19 time series modelling with the following characteristics: (i) autoregressive: to model the temporal dependencies, (ii) non-linear: to model the high variability between the different regions, (iii) to be able to incorporate exogenous data that affect the progress of the virus. Deep learning approaches are adopted here due to their inherit capability to model such scenarios [5].

This work aims at testing the efficacy of different deep learning models as tools for health care agencies to take data driven decisions in their interventions towards minimising a COVID-19 outbreak. The aim is to create tools that inherently take into account policy interventions, combine them with the current epidemiological status, and effectively provide quantifiable predictions that will help health authorities devise optimal action plans. Generalisation of the models, so that it will be able to work across borders and cover larger areas, is also very critical, as it will allow the transfer of knowledge between jurisdictions, enabling an as early as possible data driven response to the outbreak. To this end, two models are presented here, focusing on the human and resource costs of a COVID-19 outbreak, respectively. The first model predicts the new cases per million and deaths per million for the next seven days, while the second model predicts intensive care units (ICU) patients and the hospitalized (HOSP) patients for the next seven days. Taking into account the non-linearity and time-sequence of the dataset, various deep learning models are used, combining recurrent and/or convolutional structures to the non-linear character for both models. In particular, our models utilize 1D convolutional, recurrent neural network (SimpleRNN), long short term memory (LSTM), and gated recurrent unit (GRU) schemes. The models are being trained on a total of 12 European Union countries using the time period from 1 January 2020 to 15 September 2021. The estimation of the accuracy and the comparison of the methods were achieved using the Maximum Error (MaxError), the Root Mean Squared Error (RMSE), and the Mean Absolute Error (MAE). For better visualization, the predicted values of the test dataset were scattered in QQ-Plots, as well as, plotted as curvatures along with the real values.

The contributions of the paper are summarized below:

- Implementing a robust deep learning model with global coverage that approximates the non-linear character of the multi-variable model of COVID-19 evolution. Each country has different timeseries data related to the COVID-19 pandemic. These timeseries do not follow the same distribution, and it is thus challenging to create a unique model that incorporates all this information into a unique model, achieving good performance for every single country. The contribution of our model is that except for the timeseries cases per country, we have added additional features in our analysis to help the model gain the domain knowledge for each country and boost the performance of the deep learning model;
- Aiding policy makers and researchers in understanding the evolution of the COVID-19 pandemic and thus acting as a supporting tool for health management. In literature, there are various techniques for COVID-19 cases and deaths predictions, however, techniques that are applied for hospitalizations and ICU management are limited, yet they are the most important for crisis management scenarios. This study proposes models that predict all the above information at the EU level;
- Assessing the impact of the policy measures on the COVID-19 progression. Policy measures are incorporated as input variables into the model. Their variability and the different response strategies are included in the model and affect the COVID-19 progression.

## 2. Related Work

Adding intelligence in the health management related processes has been a very active research trend lately with researchers proposing data-driven adaptations from individual processes such as blood donations [6], to full-scale platforms that encompass the work of multiple organisations from various levels of health service provisioning [7]. Currently, there are various studies that propose predictive models based on mathematical modelling, infectious disease models, and machine learning models that are designed to predict the trend and patterns of COVID-19 progression [8]. Predicting the way diseases spread in different societies has thus far been documented as one of the most important tools for control strategies and policy-making during a pandemic. One of the most effective time-series methods lies in ARMA (Auto-regressive moving average) [9] and ARIMA (Auto-regressive integrated moving average) [10] modelling approaches in the COVID-19 forecasting studies. In literature, there are many country-based applications that use ARIMA models in the COVID-19 pandemic [11,12,13] and provide satisfactory results for COVID-19 prediction. However, we should highlight that ARIMA models are performed at country level, creating separate models per country and they do not adopt a unified approach. Thanks to the increased number of COVID-19 timeseries data with daily records at worldwide level, deep learning architectures for timeseries modelling have recently gained increased interest from the scientific community. The researchers use deep learning methods to model the temporal dependencies in timeseries data related to COVID-19. Recurrent neural networks (RNN) and their variants, such as Long short term memory (LSTM), Gated Recurrent Unit (GRU), and bidirectional LSTM networks are explored for modelling COVID-19 timeseries data [14,15,16,17,18]. In particular, Arko Barman in his study [19], proposed an LSTM model and comparatively analyzed its performance, using traditional ARIMA methods, for forecasting the number of confirmed COVID-19 cases. Barman’s study showed that the LSTM methods slightly underestimated while the ARIMA methods slightly overestimated the numbers in the forecasts, although both methods can be used in time series analysis for forecasting the COVID-19 cases. Chimmula et al. [20] implemented in their work a deep learning model using LSTM methods to forecast the future COVID-19 cases. In addition, Abbasimehr et al. [21], applied a time series model to forecast the number of infected cases using different RNN variants including LSTM and GRU models. Except for recurrent layers, convolutional layers are also applied in various studies [22]. Ketu et al. [23] proposed a hybrid CNN-LSTM deep learning model for correctly forecasting the COVID-19 epidemic across India (29 states). This model uses several convolutional layers (CNN), for extracting meaningful information and learning from a time series dataset, while also using LSTM layers to identify long-term and short-term dependencies.

In most of these studies the researchers analyse the temporal dynamics of the worldwide spread of COVID-19, assuming that the phenomenon has an autoregressive character. The main limitations in all the aforementioned studies is that they have regional character and do not take into consideration the spatial influence between the different countries. Most of the above mentioned studies create separate models for each country, such as in [9,12]. Some of them, have regional character, including their study adjacent regions. An example is the work of Rauf et al. [24], which explores the impact of COVID-19 pandemic for the near future in Pacific countries, particularly Pakistan, Afghanistan, India, and Bangladesh. An early attempt for a global pandemic model is the work of Khan et al. [25]. However in their analysis they do not include deep learning schemes but only shallow learning techniques.

An other limitation is that most of these studies do not consider additional features in their models to improve their performance. Work from Wang et al. [26] is an early attempt to consider the effects of preventive policy measures. This work adopts a rolling update mechanism that is applied to constantly update the input data of the model with the current prediction results, thus achieving robust predictions for the epidemic trends of COVID-19 using an improved LSTM deep learning method. Moreover, there are a few studies, such as the works of [27,28], which, except for COVID-19 cases and deaths time series, are also incorporating spatial information to improve the model’s performance at a country level. In particular, they identify relationships between geographical parameters such as latitude and longitude with the number of confirmed cases. However, these studies are limited in the literature, and to our knowledge there is no EU model for COVID-19 timeseries prediction with the use of deep learning schemes that incorporate additional feature information of the preventive measures and their effects on COVID-19 evolution.

## 3. Sequential Models for COVID-19 Evolution

### 3.1. Mathematical Formulation

COVID-19 spread results form complex dynamics in time and space. The proposed models predict the impact of COVID-19 transmission outcomes at a country level, spanning from moderate- to confirmed cases and hospitalizations, to severe outcomes—including ICU admissions and deaths—in a week ahead. In particular, we propose two deep learning models: (i) a deep learning model that predicts the daily cases and deaths due to the COVID-19 pandemic and (ii) a deep learning model for hospitalization and intensive care unit admissions. The deep learning models are trained using historic timeseries data of the COVID-19 spread in order to find a map that captures the complex trend of this spread. Furthermore, the proposed models fuse data about restrictions and policy response measures, achieving improved predictions in COVID-19 evolution. At a worldwide level, the effects of preventive measures, such as gatherings restrictions, have been reported. These preventive measures alter the spatial distribution of the reported cases across countries and differentiate the spread of the virus. Modelling analysis of COVID-19 outbreaks may help national health agencies to develop response plans.

#### 3.1.1. A Non-Linear Sequential Model to Predict Daily Confirmed Cases and Deaths Due to COVID-19 Pandemic

Our model leverages the temporal character of COVID-infections and mortality rates. Also, the model fuses information about the policy measures among with the timeseries regarding the spread of the virus at country level. Table 1 presents the list of input variables.

In Equation (1), f(·) is modelled as a non-linear relationship between the inputs and the outputs of the model:

(1) $$\begin{array}{c} {\hat{y}}_{NewCasPerMil}\left(t\right),{\hat{y}}_{NewDeaPerMil}\left(t\right)=f(x_{SchClo}\left(t\right),x_{WorClo}\left(t\right),x_{CanPubEve}\left(t\right),x_{ResGat}\left(t\right),\\ x_{CloPubTra}\left(t\right),x_{StaHomReq}\left(t\right),x_{PubInfCam}\left(t\right),x_{ResIntMov}\left(t\right),\\ x_{IntTraCon}\left(t\right),x_{FacCov}\left(t\right),x_{NewCasPerMil}\left(t\right),x_{NewDeaPerMil}\left(t\right))+\epsilon \end{array}$$

where the term ${\hat{y}}_{NewCasPerMil}$ stands for the predicted confirmed cases, whereas the term ${\hat{y}}_{NewDeaPerMil}$ is for the predicted deaths. The proposed model also includes the restrictions and lockdown measures that each country applied.

**Figure 1:**
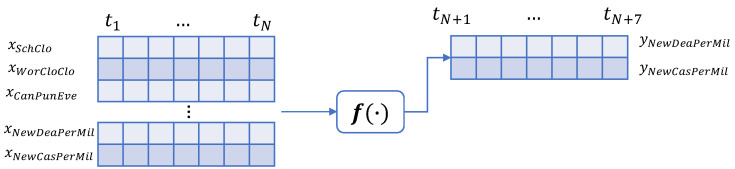
A non-linear sequential model to predict daily confirmed cases and deaths due to the COVID-19 pandemic.

As indicated in Figure 1, the input to the model is a stack of sequences. Each sequence stands for a different variable. Every element in a sequence shows the daily value of the variable covering a time period of a week, thus in each sequence there are seven elements. The model predicts two sequences each time spanning a week, one for the daily confirmed cases and another for the estimated daily deaths. The model predicts daily cases and deaths over the next week.

#### 3.1.2. A Non-Linear Sequential Model to Predict Intensive Care Admissions and Hospitalizations due to COVID-19

The COVID-19 pandemic put a massive strain on hospitals, thus, tools for guiding hospital planners in resource allocation during the pandemic are necessary. Here, we propose a machine learning model to predict the intensive care requirements for a fixed number of days into the future.

In Equation (2), g(·) is modelled as a non-linear relationship between the hospitalization and severe hospitalization of the COVID-19 patients with the restrictions and lockdown measures that each country applied.

(2) $$\begin{array}{c} {\hat{y}}_{IcuPatPerMil}\left(t\right),{\hat{y}}_{HosPatPerMil}\left(t\right)=g(x_{SchClo}\left(t\right),x_{WorClo}\left(t\right),x_{CanPubEve}\left(t\right),x_{ResGat}\left(t\right),\\ x_{CloPubTra}\left(t\right),x_{StaHomReq}\left(t\right),x_{PubInfCam}\left(t\right),x_{ResIntMov}\left(t\right),\\ x_{IntTraCon}\left(t\right),x_{FacCov}\left(t\right),x_{IcuPatPerMil}\left(t\right),x_{HosPatPerMil}\left(t\right))+\epsilon \end{array}$$

where the term ${\hat{y}}_{IcuPatPerMil}$ stands for the predicted intense care unit admissions (severe cases), whereas the term ${\hat{y}}_{HosPatPerMil}$ is for the predicted hospitalizations. The proposed model also includes the restrictions and lockdown measures that each country applied.

**Figure 2:**
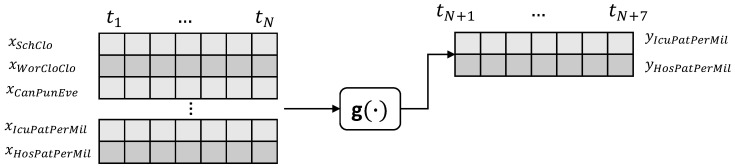
Daily hospitalization and intensive care unit submissions due to the COVID-19 pandemic.

As indicated in Figure 2, the input to the model is a stack of sequences. The model predicts two sequences each time, spanning a week; one for the daily admissions in ICUs and another for the estimated daily hospitalization.

### 3.2. Approximating Non-Linear Relationships Using Deep Learning Architectures

Equations (1) and (2) describe two non-linear relationships that cannot be easily calculated. However, a neural network can approximate such relationships in a way that minimises the error [29]. Our model maps the input sequences to an output sequence using a nonlinear approximation function. Our model adopts the so-called sequence-to-sequence (Seq2seq) modelling framework. Seq2seq models is a family of machine learning algorithms that map a sequence into another sequence. They have been originally applied in machine translation applications [30], but recently they have been adopted in a wide range of timeseries problems [31]. By utilising a Seq2seq modelling approach we are able to model the spread of the virus not only for the next day but for a sequence of days in a time period of a week. Our method uses deep learning structures to model non-linearity and to map the input sequence to a vector of a fixed dimensionality, and then applies layers to decode the target sequence from the vector.

#### 3.2.1. Recurrent Neural Networks

Recurrent models are designed to learn patterns in temporal sequences. Recurrent neural networks adopt the structure of the standard multi-layer perceptron. The difference of an RNN with a traditional neural network model is that, RNNs allow connections between hidden units associated with a time delay, thus allowing to model temporal dependencies. The RNN model approximates a non-linear operation between the inner product of the recurrent weights and output values of other neurons as well as the weighted inputs of the model.

#### 3.2.2. Long Short Term Memory Networks

**Figure 3:**
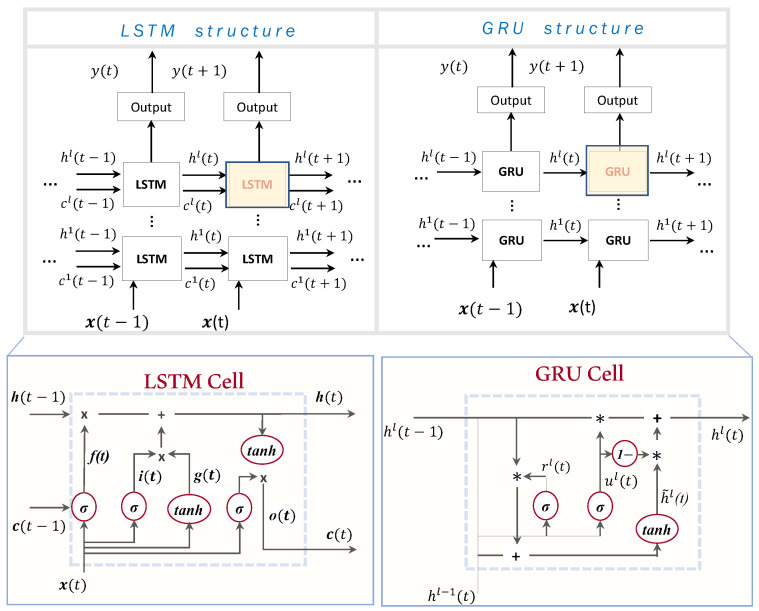
Comparison between the LSTM and GRU structures.

LSTM structure is introduced to improve the vanishing and exploding gradient problems that appear in RNN models. The single neuron of the RNN structure is replaced by the memory cell, which is a more complex structure compared to RNN’s single neuron [32]. The memory cell contains the following mechanisms: (i) the forget gate f(t), (ii) the input gate i(t), (iii) the cell candidate g(t) and (iv) the output gate o(t), as shown in Figure 3.

During the learning process, a non-linear relation to the inner product between the input vectors and weights is applied. The sigmoid σ(·) and the hyperbolic tangent function tanh(·) are the most commonly used activations. The role of the gate f(t) is to forget the unnecessary information and to separate the worth-remembering information from the useless one [33]. Input gate i(t) keeps irrelevant information from being applied in future steps, thus improving the estimation of the output values. Cell candidate g(t) activates the respective state, using the tanh activation with the range values between −1 and 1. Output gate o(t) regulates whether the response of the current memory cell is “significant enough” to pass the information to the next cell.

#### 3.2.3. Gated Recurrent Networks

The GRU network is a different variant of the wider class of the recurrent neural networks and is a simpler form compared to the previously mentioned LSTM. As shown in Figure 3, GRU has two gates that are called the *reset* gate $r^{l}\left(t\right)$ and the *update* gate $u^{l}\left(t\right)$. The reset gate $r^{l}\left(t\right)$ mechanism determines how much of the past information to forget, whereas the update gate $u^{l}\left(t\right)$ decides the worth-remembering information of the previous time steps that should pass in the future states.

#### 3.2.4. Hybrid Networks

**Figure 4:**
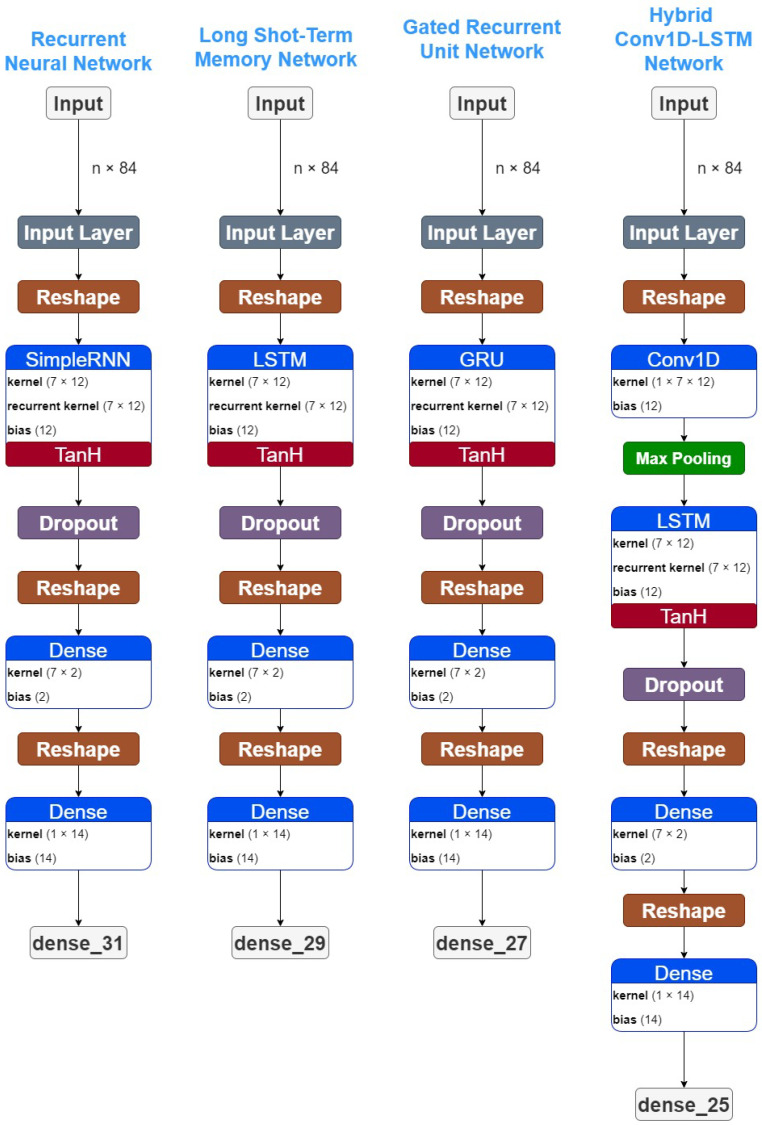
Deep learning architectures for COVID-19 predictions. The notation *n* is used for the *n*-th input that is ingested into the model.

Recurrent layers model the temporal dependencies and the sequential patterns for tasks dealing with timeseries modelling. Here, we further discuss the hybrid architecture that integrates recurrent and convolutional layers. At first, each sequence vector passes through a convolution layer with feature transformation parameters, mapping the initial input vector to a fixed-length representation vector. The output vector of the convolution layer is then passed into a recurrent sequence learning module. The core of our approach is depicted in Figure 4.

### 3.3. The Proposed Deep Learning Architectures

The case specific topology, for the above-described DL architectures is described in Figure 4. All proposed schemes utilize a 12 (features) × 7 (timestep) = 84 sequential input. Then, the recurrent layers follow, as described above. In the case of the hybrid Conv-LSTM model, a one dimensional convolutional layer precedes the recurrent ones. A dropout layer is adopted during training, which relies on stochastically “dropping out” neurons during training in order to avoid overfitting. The dense layer with a linear function following another dense layer with linear function is applied after the feature learning and the recurrent layers to perform regression.

For the purpose of this research, a hyper-parameter tuning analysis was performed for the proposed architectures (Figure 4). Multiple experiments were implemented. The accuracy of these experiments were significant low, however these experiments indicated a selective trend for the parameters. Thus, the proposed models were built using the most selected parameters of these results.

In addition, the architecture needed a Reshape Layer in the start of the model to transform the shape of the input tensor in an acceptable shape for the next layers, as well as at the end of the model to reshape the output tensor into a more sophisticated shape. To be more precise, the Input shape of (*n* × 84) needs to be reshaped as (*n* × 7 × 12), which is the correct shape for a time-series analysis. Accordingly, the output Reshape is needed to transform the (7 × 2) shape into a (1 × 14) shape, which is the desirable output shape for further analysis. The code is available at: https://github.com/JohnCrabs/CrabsMLearning (accessed on 20 March 2022).

## 4. Experimental Results

### 4.1. Dataset Description and Performance Evaluation Metrics

The COVID-19 dataset has been downloaded from Our World in Data, which is updated regularly [34]. The performance metrics are the Mean Absolute Error (MAE) and the Root Mean Squared Error (RMSE). These metrics are defined as follows:

(3) $$\mathrm{MAE}=\frac{\sum_{n}|{\hat{y}}_{i}\left(t\right)-y_{i}\left(t\right)|}{n}$$

(4) $$\mathrm{RMSE}=\sqrt{\frac{\sum_{n}{\left({\hat{y}}_{i}\left(t\right)-y_{i}\left(t\right)\right)}^{2}}{n}}$$

where *y* is the outcome of the deep learning models and *t* is the time instance and *n* is the whole time period of the measurements. The i− index stands for the values of the daily cases (i=NewCasPerMil) and the values for the daily deaths (i=NewDeaPerMil) in the case of the first model. As regards the second model, the index represents either the daily hospitalizations (i=HosPatPerMil) or the intense care unit admissions (i=IcuPatPerMil). In the MAE metric, the errors between the prediction and ground truth values are equally weighted and averaged over the whole set, whereas RMSE is more sensitive to large errors. Both metrics are selected because they are commonly used evaluation metrics in regression based models.

The COVID-19 dataset has been downloaded from the “Our World in Data” website [35]. This dataset contains the daily mitigation measures of COVID-19 in a scale of 0 (no measure) to 4 (the strictest form of this measure), worldwide, divided in multiple files. In addition, this dataset provides the daily COVID-19 cases, deaths, ICU patients, hospitalized patients, and other useful numeric information (e.g., population, population density, etc.). The full dataset is, also, available on GitHub https://github.com/owid/covid-19-data/tree/master/public/data (accessed on 20 March 2022).

For the purpose of this work, the multiple files were merged into a single dataset, using the country and date as the primary and secondary merging columns. As the next step, the dataset was divided into five smaller datasets, using the continent information (Africa, Asia, Europe, North America, South America and Oceania). The Europe dataset, which contains 44 countries in total, was used in this research.

Further data processing includes the calculation of New Cases per Million, New Deaths per Million, ICU-Patients per Million, and HOSP-Patients per Million, using the daily columns and the population column. For the model creation, we used the parameters described in Table 1 and Table 2, which were normalized. Finally, the dataset was divided into training-validation and test subsets. The training-validation subset was composed of 80% of the final selected dataset, while the remaining 20% was used as the test subset. As a cross-validation test, we selected 10% of the training-validation subset.

The implementation was applied for the full European dataset, in order to perform the evaluation of these models at an EU level. Except for the proposed deep learning models, in addition, we have tested the results with traditional epidemiological approaches. Thus, Arima models were created using this dataset to evaluate the performance at an European scale.

### 4.2. Case Study 1: New Cases per Million and New Deaths per Million

The prediction results for the daily cases and deaths during the pandemic period is a significant factor for policy makers and health caretakers. Case study 1 investigates the estimation of future new cases and deaths, depending on the current policy measures, as well as the previous reported values of cases and deaths. Table 3 and Table 4 summarise the estimated performance errors per method for the test set. Our results are shown for each of the 12 EU countries. The smaller error values indicate better results. The best values per method and per country are highlighted in bold.

#### 4.2.1. New Cases per Million Analysis

Table 3 presents the errors for new cases per million for each country per method. SimpleRNN appears to have smaller error values in terms of MAE for Belgium, Denmark, Estonia, France, Ireland, Italy, Netherlands, and Portugal. LSTM has the minimum error compared to the other techniques for Austria, Germany, and Romania. As regards the RMSE evaluation metric, this metric is more sensitive to large errors when compared to MAE, which measures the average magnitude of the errors in a set of predictions. Denmark, Estonia, France, Ireland, Italy, Netherlands, and Portugal show the minimum RMSE values for SimpleRNN network. The GRU network shows the minimum RMSE values for Finland and Belgium. For the rest countries LSTM shows the minimum RMSE error values. The best fitted countries appear to be Austria, Romania, and Italy, while the Netherlands and France estimations indicated the highest errors.

**Figure 5:**
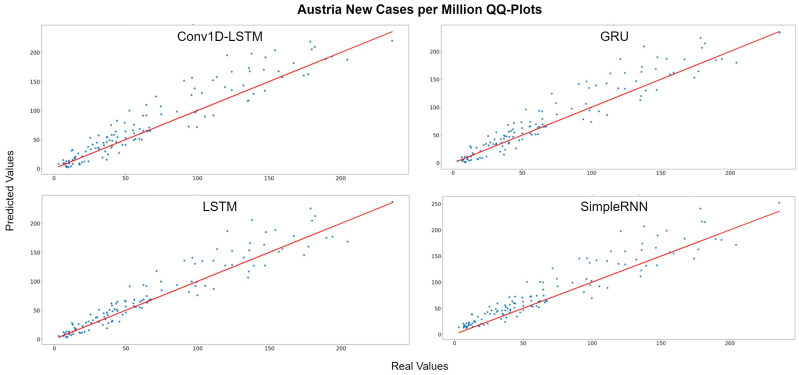
Austria new cases per million QQ-Plot for each method.

**Figure 6:**
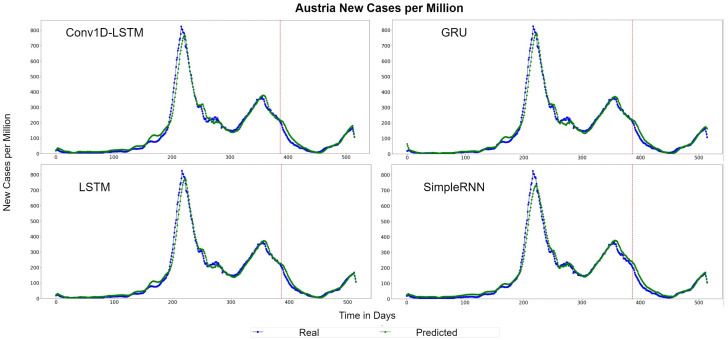
Austria’s new cases per million real (blue line) and predicted (green line) values for each method, using training, validation and test datasets. The red line divides the train-validation (right size) and the test (left side) datasets.

Figure 5 depicts the Quantile–Quantile (Q-Q) plots for Austria. The Q-Q plots is a graphical method used to compare the distributions of the real ground truth data and the estimated data from the deep learning structures. Assuming that these two distributions are similar, the points in the Q-Q plot will lie on the diagonal line, which is indicated with a red colour in Figure 5. The less the distance of the data points from the red line is, the better the accuracy. For all methods, the dominant trend is that the estimations are more accurate when the number of cases are lower (closer to 0). The QQ-Plots also indicate that the best results for Austria was observed in GRU and LSTM methods because the scatter-plot is closer to the red line. The same observation also appears also in Figure 6, where the real (blue lines) and predicted (green lines) values of new cases per million for each method are presented, using the whole time-series dataset for Austria. The red hashed line divides the train-validation (left side) and the test (right side) sets.

**Figure 7:**
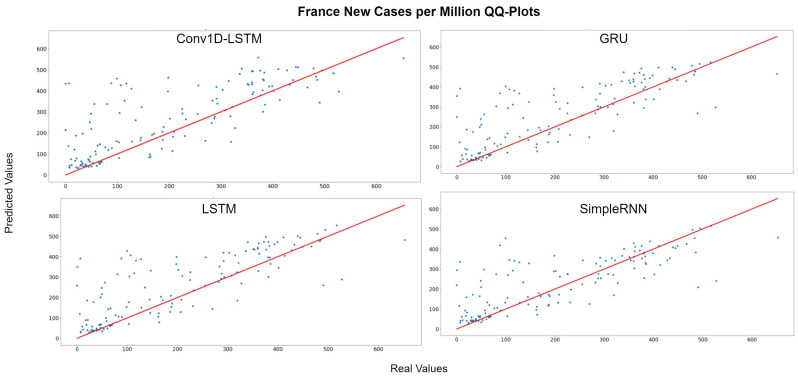
France new cases per million QQ-Plot for each method.

**Figure 8:**
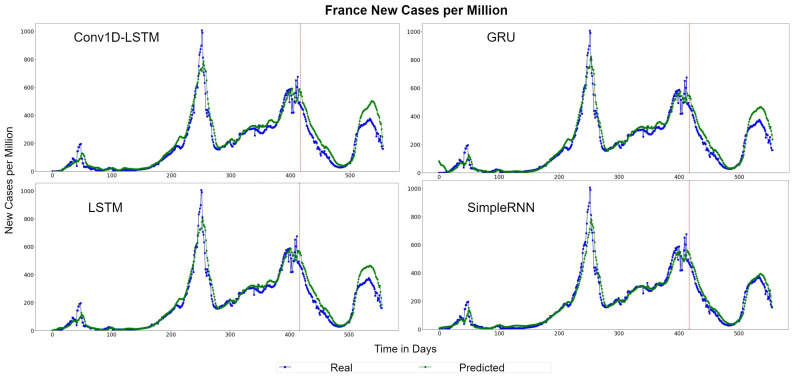
France new cases per million real (blue line) and predicted (green line) values for each method, using the training, validation, and test datasets. The red line divides the train-validation (right size) and the test (left side) datasets.

Figure 7 depicts the QQ-Plot of the new cases per million of each method for France for the test set. QQ-Plot analysis indicates that the test set range is between 0 and 600 cases per million and the higher predicted values appear to be closer to the red line (real values). Figure 8 depicts the new cases per million real (blue line) and predicted (green line) values of each method for the full dataset. The curve of the real values for France has a lot of peaks (outliers), which affects the overall accuracy of the models. However, the deep learning models recognize the general trend of the time-series and the predictions are smoother. SimpleRNN estimations were better than the estimations of the other methods, in this case.

#### 4.2.2. Daily Deaths per Million Analysis

Table 4 describes the errors for the daily deaths per million, for each country per method. The MAE and RMSE errors show as smaller values for the GRU method in the cases of Austria, Belgium, France, Germany, Ireland, Italy, Netherlands, and Portugal. Overall, Denmark shows the best performance. In this case, the model predicts the daily deaths per million with an error smaller than 1 person (${\mathrm{MAE}}_{\mathrm{SimpleRNN}}=0.44,{\mathrm{RMSE}}_{\mathrm{SimpleRNN}}=0.49$). As regards the other countries, Italy, Finland, and Austria appear to have the best results, while Ireland and Romania have the highest errors.

**Figure 9:**
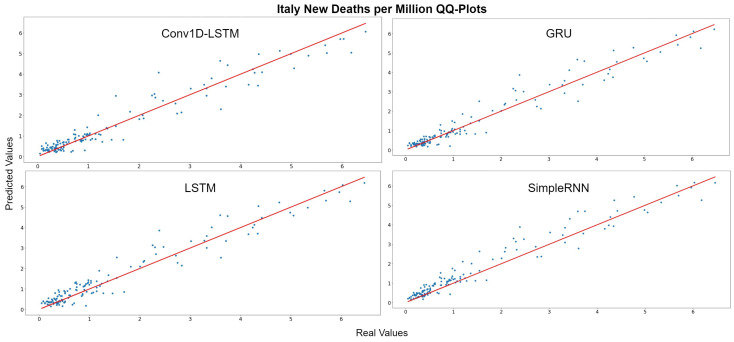
Italy’s new deaths per million QQ-Plot for each method.

**Figure 10:**
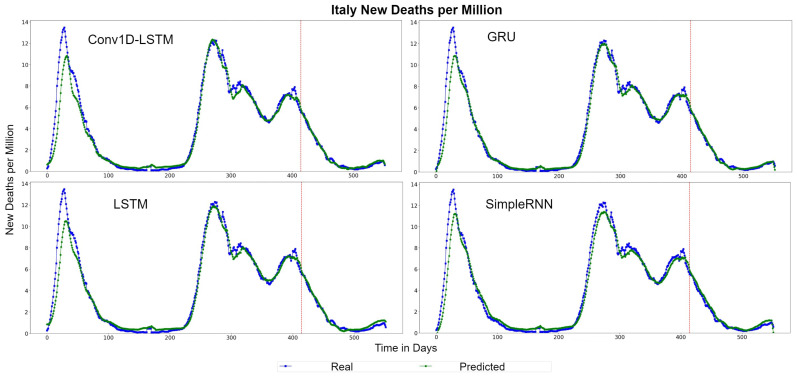
Italy’s new deaths per million real (blue line) and predicted (green line) values for each method, using the training, validation and test datasets. The red line divides the train-validation (right size) and the test (left side) datasets.

The QQ-Plots of daily deaths per million for the test set period, for each method, for Italy are displayed in Figure 9. As shown in the x- and y- axis, the values of the daily deaths per million have a range between 0 and 6 deaths during the test set period. Figure 10 shows the real (blue line) and predicted (green line) values for the whole period, including the training-validation period as well as the test period. These two periods are divided in the diagram with a vertical red-dotted line. For all the examined methods, the predicted line follows the general trend, however it is observed that all the models underestimate the deaths in their highest values. The GRU and SimpleRNN models seems to be slightly better than the other methods, especially in the predictions of the test dataset.

**Figure 11:**
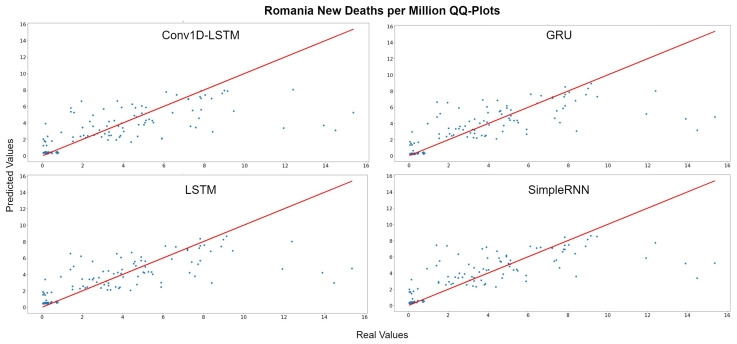
Romania’s new deaths per million QQ-Plot for each method.

**Figure 12:**
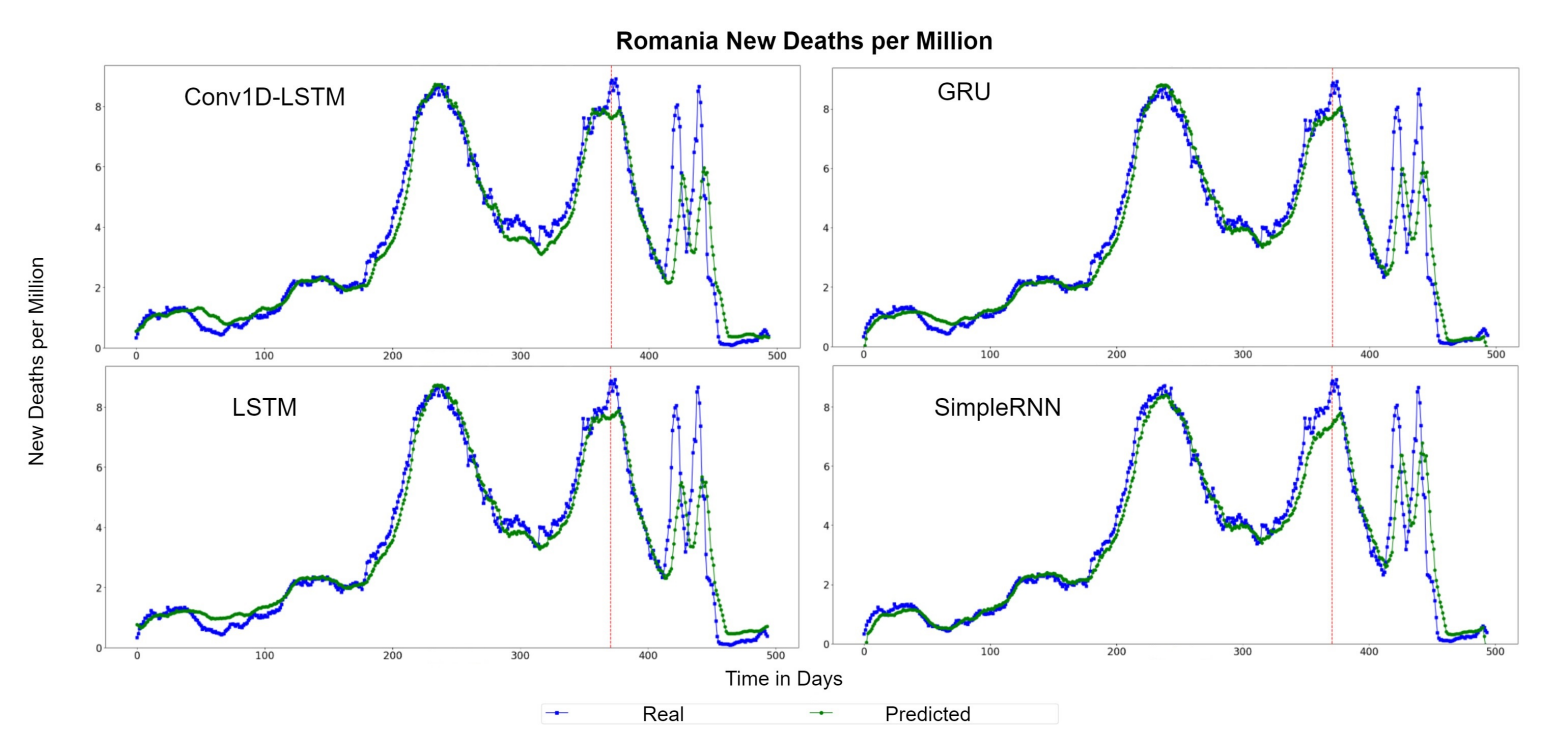
Romania’s new deaths per million real (blue line) and predicted (green line) values for each method, using the training, validation, and test datasets. The red line divides the train-validation (right size) and the test (left side) datasets.

The QQ-Plots for Romania, as shown in Figure 11, reinforces our previous observation, in which all models underestimate the higher values. Figure 12 depicts the real (blue line) and predicted (green line) values of Romania for the full dataset. GRU and SimpleRNN estimations are better than Conv1D-LSTM and LSTM. However, the overall accuracy in Romania is low, due to the high values of deaths per million.

### 4.3. Case Study 2: ICU Patients Admissions and Hospitalizations per Million of People

Intensive care unit patients and hospitalized (HOSP) patients can also be used, as metrics in a pandemic. The estimation of these two metrics can help the policy makers, health takers, and the government, either to reinforce the health system beforehand, or to take stricter mitigation measures. This can help to unload or prevent the pressure inside the hospitals, which can minimize the death rate.

#### 4.3.1. ICU-Patients per Million Results

Table 5 describes the performance errors for ICU admissions of the patients per million of population, for each country per method. Due to the scale of the dataset, the errors have low values. In this case, GRU and LSTM appears to have the lowest errors in most cases. The Denmark, Finland, and Portugal estimation scores are better, whereas Estonia and Romania have the highest errors; however, there is an accurate overall performance.

**Figure 13:**
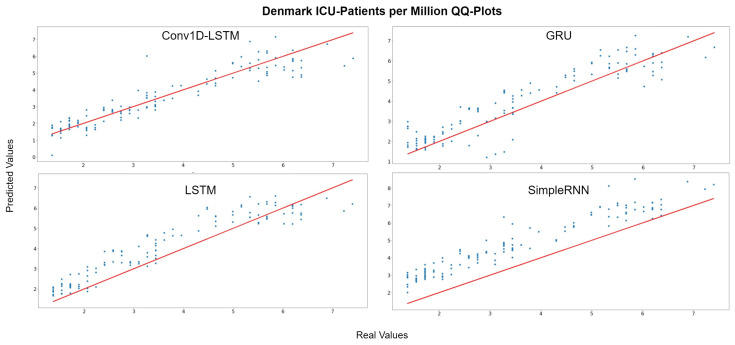
Denmark ICU-Patients per million QQ-Plot for each method.

**Figure 14:**
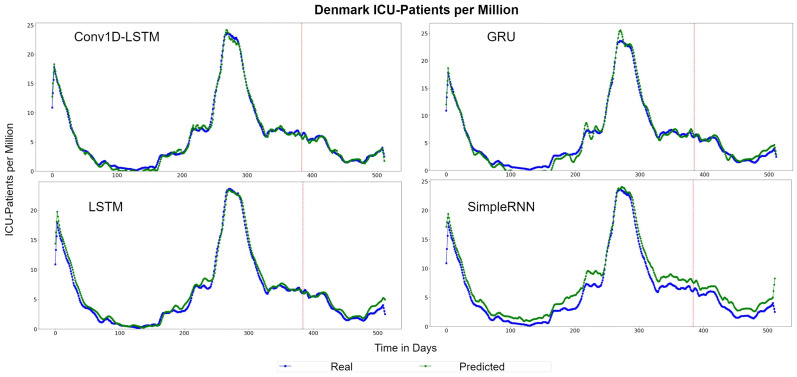
Denmark ICU-Patients per million real (blue line) and predicted (green line) values for each method, using the training, validation, and test datasets. The red line divides the train-validation (right size) and the test (left side) datasets.

Figure 13 depicts the QQ-Plots for ICU-Patients per million, for each method for Denmark. Conv1D-LSTM is the best method, in this case, as it is also shown in Table 5 with bold. GRU, LSTM, and SimpleRNN appear to have an offset. Figure 14 depicts the real (blue line) and predicted (green line) values for the full dataset. Conv1D-LSTM estimations are almost the same as the real values. Also, it is observed that the GRU and LSTM methods follow the general trend of the curve, with a little delay. SimpleRNN is in this case is the worst model; however, the predicted values, in addition to the time delay, appear to be overestimated by an inversely proportional amount. Thus, the lower the real value, the higher the difference (error) between the real and estimated value.

**Figure 15:**
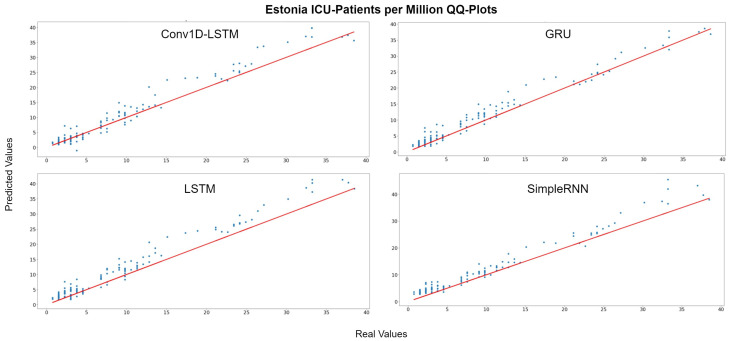
Estonia ICU-Patients per million QQ-Plot for each method.

**Figure 16:**
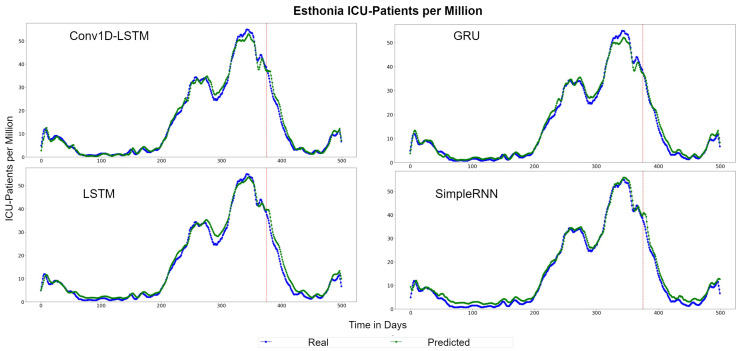
Estonia ICU-Patients per million real (blue line) and predicted (green line) values for each method, using the training, validation and test datasets. The red line divides the train-validation (right size) and the test (left side) datasets.

The depicted QQ-Plots for Estonia presented in Figure 15. We can observe a trend, where, the lower the value, the better the result will be. Additionally, in this case, the GRU scatter-plot is closer to the red line. However, an offset is observed at all methods. Figure 16 depicts the real (blue line) and the predicted (green line) values. In this figure, it is clearer that a time delay (offset) exists. Thus, when in reality the curvature changes at time $t_{1}$, the prediction curve follows after some days. In the SimpleRNN method, an overestimated inversely proportional vertical offset is also observed.

#### 4.3.2. HOSP-Patients per Million Results

Table 6 presents the errors for the hospitalized patients per million of population, for each method. In most cases, LSTM appears to have the smaller error values, while SimpleRNN is the second best. RMSE and MAE scores are almost equally, which means smooth curvatures, without many outlier values. The countries with the highest errors (lower accuracy) appear to be France, Finland, and Estonia, while the estimations for Austria, Denmark, and Romania resulted in small errors (higher accuracy).

**Figure 17:**
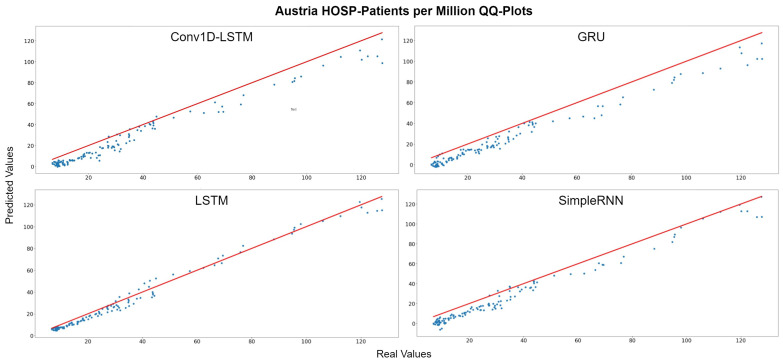
Austria HOSP-Patients per million QQ-Plot for each method.

**Figure 18:**
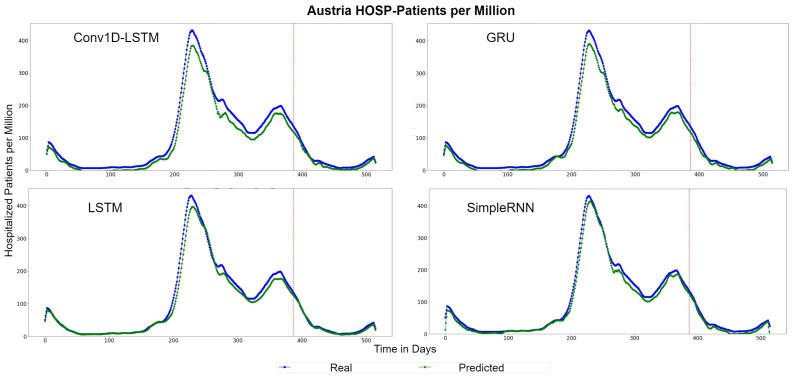
Austria’s HOSP-Patients per million real (blue line) and predicted (green line) values for each method, using the training, validation, and test datasets. The red line divides the train-validation (right size) and the test (left side) datasets.

Figure 17 depicts the HOSP-Patients QQ-Plots of Austria, for each method. All models underestimate the estimations by a vertical fixed amount. The best method appears to be LSTM, where the amount is smaller. Similar observation can be seen in Figure 18, which depicts the real (blue lines) and predicted (green lines) curves. In this figure, we can also observe that SimpleRNN predicted the higher values of the train-validation set better than the other methods, while LSTM predicted the lower values better.

**Figure 19:**
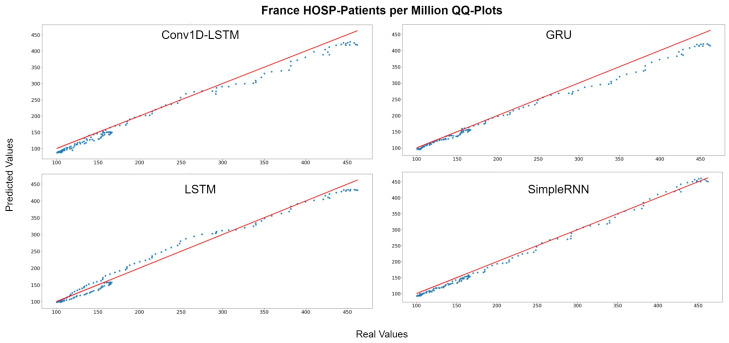
France HOSP-Patients per million QQ-Plot for each method.

**Figure 20:**
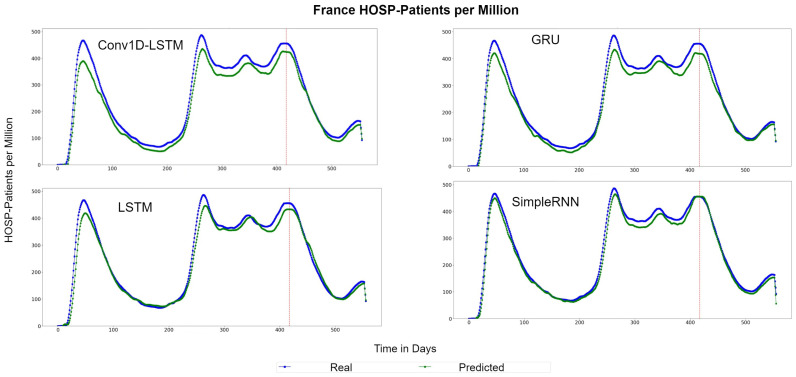
France HOSP-Patients per million real (blue line) and predicted (green line) values for each method, using the training, validation, and test datasets. The red line divide the train-validation (right size) and the test (left side) datasets.

The example for France indicates similar observations. The predicted values are underestimated by a vertical fixed amount in Conv1D-LSTM, GRU, and SimpleRNN methods, as shown in Figure 19, which depicts the QQ-Plots. LSTM seems to underestimate in some cases and to overestimate in other cases, however the distance (MAE) from the true values (red line) is between a small range. Figure 20 depicts the real (blue line) and predicted (green line) curves. SimpleRNN and LSTM seems to be the two best methods, however, SimpleRNN is more accurate in higher values and LSTM is better in lower values.

### 4.4. Average Global European Model Results

Table 7 summarizes the results of this work, including the EU countries, for the period of 1 March 2020 to 15 September 2021. The average RMSE and MAE errors are presented in Table 7. For the predictions of New Cases per Million and ICU-Patients per Million, the GRU models appear to have the best results. In the case of New Deaths per Million and Hosp-Patients per Million, the LSTM models indicate slightly better results then GRU. The Conv1D-LSTM models have the highest errors, compared to the other deep learning approaches, while SimpleRNN is close enough.

For completeness of the research, the comparison between the deep learning approaches and traditional methods is needed. The ARIMA approach is one of the most common traditional epidemiological approaches for time series analysis and forecasting. In this work, ARIMA models were trained using the same datasets and tested using the same complete dataset, as described in the previous paragraph. Table 7 indicates that the ARIMA models were unable to achieve sufficient predictions at the aggregated EU level, and performed poorly, compared to the proposed deep learning approaches. This may be due to the complexity of the transmissibility of the COVID-19, and due to the fact that the spread of the virus varies from one country to another. The ARIMA model cannot effectively represent the non-linear behaviour of these variables, thus the ARIMA models appear to perform poorly in comparison with the deep learning models.

## 5. Conclusions

In this work, we propose two sequential models to represent the COVID-19 evolution. The first model predicts the daily cases and deaths due to COVID-19 virus and the second one predicts the daily data on hospitalizations and intensive care (ICU) admissions due to COVID-19 with a weekly range. These models incorporate information about the restrictions and policy responses applied due to COVID-19. In our experiments, we test four different deep learning methods: (a) Conv1D-LSTM, (b) GRU, (c) LSTM, and (d) SimpleRNN. In the first case study, the results showed that all four methods can be used for the estimation of future cases and deaths, however SimpleRNN gave better results than the other methods for most of the countries. In the second case study, LSTM is preferable, because it performed good in both ICU admissions and hospitalized patients predictions.

Our study introduces two models with generalization abilities. The experimental results are presented for various different countries in EU and show that in most cases the model achieves good performance. In our future work, we will further investigate the model’s scalability and applicability by incorporating additional input variables and also discuss the role of vaccinations in COVID-19 evolution.

The approach presented in this paper showcases significant advantages to classical epidemiological, or simple time-series approaches, as it is able to generalise in higher spatial granularity, and inherently takes into account interventions during the modelling and training procedures. This means that the resulting models are not only able to execute knowledge transfer procedures across jurisdictions, but are also able to quantify interventions during the prediction process, allowing policy makers and health care administrators to take the optimal course using data driven decision making. Regarding future work, explainable and interpretable machine learning models will be adopted to rank the importance of the various features in COVID-19 progression.

## Figures and Tables

**Table 1:** Description of input variables into the deep learning model for daily cases and deaths.

Index	Variable	Column Data
1	$x_{SchClo}$	School Closures
2	$x_{WorClo}$	Workplace Closures
3	$x_{CanPubEve}$	Cancel Public Events
4	$x_{ResGat}$	Restriction in Gatherings
5	$x_{CloPubTra}$	Close Public Transport
6	$x_{StaHomReq}$	Stay Home Requirements
7	$x_{PubInfCam}$	Public Information Campaigns
8	$x_{ResIntMov}$	Restrictions Internal Movements
9	$x_{IntTraCon}$	International Travel Controls
10	$x_{FacCov}$	Facial Coverings
11	$x_{NewCasPerMil}$	New Cases per Million
12	$x_{NewDeaPerMil}$	New Deaths per Million

**Table 2:** Description of input variables into the deep learning model hospitalizations and icu admissions.

Index	Variable	Column Data
1	$x_{SchClo}$	School Closures
2	$x_{WorClo}$	Workplace Closures
3	$x_{CanPubEve}$	Cancel Public Events
4	$x_{ResGat}$	Restriction in Gatherings
5	$x_{CloPubTra}$	Close Public Transport
6	$x_{StaHomReq}$	Stay Home Requirements
7	$x_{PubInfCam}$	Public Information Campaigns
8	$x_{ResIntMov}$	Restrictions Internal Movements
9	$x_{IntTraCon}$	International Travel Controls
10	$x_{FacCov}$	Facial Coverings
11	$x_{IcuPatPerMil}$	ICU-Patients per Million
12	$x_{HosPatPerMil}$	HOSP-Patients per Million

**Table 3:** Errors for New Cases per Million for each country per method. Bold values showcase the minimum error achieved in each country.

New Cases per Million						
**Methods**	**RMSE**	**MAE**	**RMSE**	**MAE**	**RMSE**	**MAE**
	Austria		Belgium		Denmark	
Conv1D-LSTM	22.25	16.23	99.31	75.02	59.71	46.60
GRU	20.49	14.85	**90.62**	69.98	60.32	46.72
LSTM	**18.80**	**13.32**	91.76	70.94	64.50	49.93
SimpleRNN	22.67	17.02	93.38	**66.95**	**46.20**	**32.51**
**Methods**	**RMSE**	**MAE**	**RMSE**	**MAE**	**RMSE**	**MAE**
	Estonia		Finland		France	
Conv1D-LSTM	76.43	55.75	36.17	26.10	121.99	82.83
GRU	61.25	44.80	**33.45**	**22.81**	108.13	73.20
LSTM	72.71	54.37	34.46	23.75	111.92	76.21
SimpleRNN	**41.90**	**32.10**	35.85	24.22	**101.62**	**67.71**
	Germany		Ireland		Italy	
Conv1D-LSTM	35.97	21.93	62.89	46.35	32.14	24.77
GRU	33.22	19.25	56.96	41.64	27.20	21.20
LSTM	**33.16**	**18.79**	62.25	45.02	26.75	20.66
SimpleRNN	37.67	24.71	**34.93**	**25.76**	**21.53**	**16.83**
	The Netherlands		Portugal		Romania	
Conv1D-LSTM	138.46	101.10	68.83	47.10	26.59	18.73
GRU	119.79	88.70	59.81	41.86	17.83	14.15
LSTM	140.93	102.03	60.46	41.34	**16.52**	**12.20**
SimpleRNN	**85.96**	**61.99**	**46.16**	**32.48**	17.22	13.35

**Table 4:** Errors for the daily deaths per million for each country per method. The minimum MAE and RMSE errors per country are in bold.

New Deaths per Million						
**Methods**	**RMSE**	**MAE**	**RMSE**	**MAE**	**RMSE**	**MAE**
	Austria		Belgium		Denmark	
Conv1D-LSTM	0.42	0.33	0.56	0.43	0.61	0.56
GRU	**0.41**	**0.32**	**0.48**	**0.37**	0.50	0.45
LSTM	0.45	0.38	0.50	0.39	0.60	0.55
SimpleRNN	0.43	0.34	0.49	0.38	**0.49**	**0.44**
	Estonia		Finland		France	
Conv1D-LSTM	0.99	0.80	0.43	0.37	0.77	0.60
GRU	**0.96**	0.77	0.44	0.37	**0.63**	**0.48**
LSTM	1.00	0.81	0.45	0.39	0.67	0.53
SimpleRNN	0.97	**0.74**	**0.39**	**0.30**	0.64	0.50
	Germany		Ireland		Italy	
Conv1D-LSTM	0.57	0.39	1.08	0.77	0.40	0.28
GRU	**0.54**	**0.36**	**1.02**	**0.66**	**0.36**	**0.25**
LSTM	0.55	0.38	1.08	0.67	0.39	0.30
SimpleRNN	0.58	0.43	1.03	0.69	0.41	0.31
	The Netherlands		Portugal		Romania	
Conv1D-LSTM	0.75	0.62	0.46	0.35	2.47	1.44
GRU	**0.68**	**0.54**	**0.39**	**0.29**	2.31	**1.30**
LSTM	0.69	0.58	0.46	0.37	2.36	1.38
SimpleRNN	0.75	0.61	0.46	0.37	**2.29**	1.33

**Table 5:** Errors for ICU admissions of the patients per million of the population for each country per method. Bold values show the minimum error achieved per country.

Intensive Care Unit Patients per Million						
**Methods**	**RMSE**	**MAE**	**RMSE**	**MAE**	**RMSE**	**MAE**
	Austria		Belgium		Denmark	
Conv1D-LSTM	1.44	1.08	2.16	1.59	**0.62**	0.46
GRU	**1.33**	**1.01**	**1.82**	**1.47**	0.74	0.60
LSTM	1.50	1.17	3.33	2.72	0.74	0.63
SimpleRNN	1.57	1.27	2.76	2.46	1.34	1.26
	Estonia		Finland		France	
Conv1D-LSTM	2.48	1.76	0.90	0.66	2.31	1.80
GRU	**2.17**	**1.65**	1.01	0.85	**1.89**	**1.60**
LSTM	2.91	2.27	**0.79**	**0.64**	3.43	2.66
SimpleRNN	2.74	2.13	2.35	2.26	2.91	2.43
	Germany		Ireland		Italy	
Conv1D-LSTM	1.92	1.51	0.92	**0.71**	**1.24**	**0.91**
GRU	1.24	1.05	1.03	0.73	1.52	1.12
LSTM	**1.21**	**0.94**	**0.91**	0.72	1.49	1.17
SimpleRNN	1.54	1.32	1.45	1.34	1.45	1.20
	The Netherlands		Portugal		Romania	
Conv1D-LSTM	1.49	1.22	**0.94**	**0.74**	3.19	2.44
GRU	1.33	1.12	1.48	1.24	**2.19**	**1.72**
LSTM	1.54	1.32	1.16	0.87	3.38	2.99
SimpleRNN	**1.32**	**1.09**	1.18	0.99	2.76	2.48

**Table 6:** Errors for HOSP-Patients per Million for each country per method. Bold values show the minimum errors per country.

Hospitalized Patients per Million						
**Methods**	**RMSE**	**MAE**	**RMSE**	**MAE**	**RMSE**	**MAE**
	Austria		Belgium		Denmark	
Conv1D-LSTM	9.29	8.18	13.23	11.30	10.67	10.05
GRU	10.25	9.15	16.57	14.18	10.67	10.14
LSTM	**4.18**	**3.52**	**5.41**	**3.94**	**5.68**	**4.72**
SimpleRNN	8.71	7.87	7.03	6.20	6.55	5.82
	Estonia		Finland		France	
Conv1D-LSTM	12.25	10.02	17.89	17.48	17.21	14.54
GRU	**9.15**	**7.35**	19.54	18.10	18.00	13.00
LSTM	15.26	11.85	4.45	3.63	13.14	10.95
SimpleRNN	13.28	8.03	**4.39**	**3.04**	**10.18**	**9.33**
	Germany		Ireland		Italy	
Conv1D-LSTM	11.01	9.74	15.65	15.16	17.26	15.49
GRU	16.56	15.19	15.18	14.70	13.60	12.13
LSTM	**3.79**	**3.50**	**5.05**	**3.49**	**8.62**	**6.69**
SimpleRNN	8.77	7.22	8.09	7.08	9.78	8.46
	The Netherlands		Portugal		Romania	
Conv1D-LSTM	10.25	8.11	16.31	15.42	10.23	8.41
GRU	12.57	9.84	14.69	13.24	11.87	9.42
LSTM	**7.63**	**6.50**	**7.30**	**6.01**	12.03	10.25
SimpleRNN	8.33	7.29	8.26	7.35	**10.02**	**7.32**

**Table 7:** Averaged RMSE and MAE errors for the global European Model. Traditional approaches, such as ARIMA, are also included. The minimum MAE and RMSE errors are highlighted with bold.

	Global European Model Average Errors									
**Country**	**Conv1D-LSTM**		**GRU**		**LSTM**		**SimpleRNN**		**ARIMA**	
	**RMSE**	**MAE**	**RMSE**	**MAE**	**RMSE**	**MAE**	**RMSE**	**MAE**	**RMSE**	**MAE**
New Cases per Million	384.70	214.86	**264.03**	**150.66**	332.36	184.50	365.94	191.47	645.13	536.47
New Deaths per Million	6.08	3.26	4.89	2.60	**4.13**	**2.28**	5.39	2.91	12.22	11.01
ICU-Patients per Million	8.62	5.94	**6.92**	**4.80**	11.90	8.35	10.80	7.23	41.09	36.75
HOSP-Patients per Million	89.05	57.46	87.53	56.05	**85.28**	**50.81**	104.44	60.11	318.92	282.08

## Data Availability

Not applicable.

## Abbreviations

The following abbreviations are used in this manuscript:

ARIMA	Autoregressive Integrated Moving Average
Conv1D	One Dimension Convolution
LSTM	Long Short Term Memory
GRU	Gated Recurrent Units
SimpleRNN	Simple Recurrent Neural Network
MAE	Mean Absolute Error
RMSE	Root Mean Square Error
ICU	Intensive Care Unit
HOSP	Hospitalized
